# Twelve-month results for a randomized sham-controlled effectiveness trial of an in-home skills-based virtual reality program for chronic low back pain

**DOI:** 10.1097/PR9.0000000000001182

**Published:** 2024-09-04

**Authors:** Todd Maddox, Liesl Oldstone, Josh Sackman, Roselani Maddox, Takisha Adair, Kelsey Ffrench, Charisse Sparks, Beth D. Darnall

**Affiliations:** a AppliedVR, Inc., Van Nuys, CA, USA; b Inspire Medical Systems, Inc, Golden Valley, MN, USA; c Department of Anesthesiology, Perioperative and Pain Medicine, Stanford University School of Medicine, Palo Alto, CA, USA

**Keywords:** Virtual Reality, Chronic pain, Cognitive behavioral therapy

## Abstract

**Introduction:**

Low-risk, accessible, and long-term effective nonpharmacologic behavioral interventions for chronic low back pain (cLBP) are needed. Pain education and cognitive behavioral therapy (CBT) are recommended first-line treatments, but access is poor, treatment effectiveness is variable, and long-term effectiveness is inconsistent. In-home virtual reality (VR)-delivered therapies might address these shortcomings because therapeutic content can be delivered in a consistent and quality-controlled manner.

**Objective:**

To determine whether a 56-session, self-administered in-home, Skills-Based VR program for cLBP (RelieVRx) yields long-term reductions in pain intensity and pain interference 12 months posttreatment in a large demographically diverse and clinically severe real-world sample.

**Methods:**

Participants were 1,093 demographically diverse individuals with self-reported nonmalignant cLBP >3 months duration and average pain intensity and interference scores >4/10. Participants were randomized to Skills-Based VR or active Sham, and data were collected from January 31, 2022 to October 31, 2023. Pretreatment to 12-month posttreatment analyses were conducted.

**Results:**

From baseline to 12 months posttreatment, Skills-Based VR reductions for average pain intensity (1.7 ± 2.1) and pain interference (1.9 ± 2.3) were robust and significantly greater than those found for Sham. More than half of Skills-Based VR participants reported at least a 2-point reduction in pain intensity, pain interference, or both at 12 months posttreatment.

**Conclusions:**

A standardized, in-home Skills-Based VR therapy is effective for reducing pain intensity and pain interference, and these effects are maintained to 12 months posttreatment.

## Abstract 2

Robust reductions in pain intensity and pain interference were maintained at 12-months posttreatment for a Skills-Based virtual reality program for chronic low back pain.

Supplemental Digital Content is Available in the Text.

## 1. Introduction

Chronic low back pain (cLBP) affects one-third of adults globally and imparts substantial suffering, disability, and cost to patients, families, society, and payors. Community-based clinicians continue to struggle in their quest for effective and accessible non-pharmacologic options for cLBP. With opioid prescribing decreasing, the US Centers for Disease Control and Prevention (CDC) and US Centers for Medicare and Medicaid Services (CMS)^12,41,46^ call for low-risk, accessible, and long-term effective nonpharmacologic behavioral interventions for cLBP. Pain education and cognitive behavioral therapy (CBT) are recommended as first-line treatments given their low risk.^4,16,37,48^ Even so, access is poor because of the multisession and therapist-led nature of CBT.^9^ In addition, effectiveness can fluctuate based on the quality of the CBT therapist and the trial methods, with the long-term effectiveness being variable at 12 months posttreatment.^10,13,22,34,47,49^

Immersive virtual reality (VR) devices address key shortcomings because self-administered therapeutic content can be delivered in a consistent, quality-controlled manner at home.^32,42–44^ The immersive nature of VR-delivered content broadly engages multiple centers in the brain in synchrony^29,32^ and can target pain processing brain regions known to be involved in cLBP^11,17,24,25,28,35,45^ that are responsive to CBT treatment.^1,39,40,50^ These include dorsolateral prefrontal cortex, orbitofrontal cortex, ventrolateral prefrontal cortex, posterior cingulate cortex, thalamus, primary motor regions, and the amygdala to name a few.^32^ [The interested reader is directed to Maddox et al. (2023) for a more detailed discussion.] Thus, VR-based therapies may provide a low-risk, accessible, long-term effective treatment for cLBP. Therapeutic programs, such as the proprietary Skills-Based VR for Chronic Pain program (RelieVRx), can be delivered in VR and combine pain education, CBT, diaphragmatic breathing, biofeedback, and mindfulness, that collectively help patients develop coping skills to address cLBP. The ease-of-use and potential for repetition in VR allows these pain coping skills to become habits that are long lasting.

Two recent double-blind, randomized, placebo-controlled trials compared the 56-session in-home Skills-Based VR program to Sham VR. In the first trial, Garcia et al.^21^ tested N = 188 community-based adults with cLBP who were homogeneous (female: 76%, non-White: 8%, high school or less: 8%, without depressive symptoms) and clinically moderate (baseline pain intensity = 5.1; baseline pain interference = 4.8, disability = within normal range; sleep disturbance = mild).^20,21^ Clinically meaningful reductions (≥2 points^15^) in pain intensity (2.2 ± 1.6) and pain interference (2.6 ± 2.3) were observed at the end of treatment for Skills-Based VR that were significantly larger than for Sham and durable up to 24 months posttreatment.^30,33^ The lack of clinical and demographic diversity among study participants limited the generalizability of results. In the second trial, Maddox et al.^31^ addressed these methodologic shortcomings by conducting a new trial in a sample of N = 1093 adults with cLBP with greater demographic diversity (female: 72%, non-White: 32%, high school education or less: 20%, depressive symptoms not excluded) and clinical severity (baseline pain intensity = 6.6; baseline pain interference = 6.2, disability = severe/completely disabled; sleep disturbance = moderate/severe). Clinically meaningful reductions in pain intensity (2.0 ± 1.9) and pain interference (2.3 ± 2.0) were observed at the end of treatment for Skills-Based VR that were significantly larger than for Sham (for a review of several VR-based chronic pain programs, see Refs. 36, 38). The present study examined the primary outcomes of pain intensity and pain interference reduction and the secondary outcomes of sleep disturbance, depression, and physical disability reduction at 12 months posttreatment relative to baseline in the randomized controlled trial by Maddox et al.

## 2. Methods

### 2.1. Study design, participants, and randomization

Full Methods for the original clinical trial by Maddox et al. were published.^31^ The trial compared the effectiveness of Skills-Based VR with Sham. The focus of this report was on the long-term treatment effectiveness at a 12-month posttreatment follow-up. The study protocol was approved by the WCG Institutional Review Board (Puyallup, WA) in December 2021 and followed the Consolidated Standards of Reporting Trials (CONSORT) reporting guidelines. Written informed consent was obtained before enrollment. Twelve-month posttreatment data were collected from April to October 2023.

Individuals with self-reported cLBP (>3 months and average pain intensity and interference of >4 for the past month on a 0–10 pain rating scale; Brief Pain Inventory^5^) were recruited through online advertisements, chronic pain organizations, and pain clinics. Exclusion criteria included any condition that prevented comfortable use of VR, cancer-related back pain, suicide ideation, current, previous, or upcoming participation in a separate randomized controlled trial or medical procedure focused on cLBP. Self-reported survey responses were used to determine inclusion and exclusion. Once consented, cLBP participants were randomized 1:1:1:1 to one of the following 4 conditions: (1) a 56-day Skills-Based VR program, (2) a 56-day Skills-Based VR program followed by a 56-day on-demand period with the same skills-based content, (3) a 56-day Sham, or (4) a 56-day Sham followed by a 56-day on-demand period with the same sham content. Because the number of VR experiences completed (out of a total of 56) was low during the on-demand period (detailed in the Results section), this report combined the 2 Skills-Based VR arms and the 2 Sham arms. Participants were blinded to treatment and remained blinded during posttreatment follow-up.

### 2.2. Patient-reported outcomes and study group interventions

Per preregistered study methods, several patient-reported outcomes were collected at baseline, end-of-treatment, and at 1, 2, 3, 6, and 12 months posttreatment. Most relevant to the current report are the Brief Pain Inventory (BPI^5^), which measures pain intensity and pain interference over the last 24 hours using a 0 to 10 numeric pain rating scale, as well as the Patient-Reported Outcomes Measurement Information System (PROMIS) short-form for depression (version 8b) (T-score range: 37.1–81.1; mild-to-moderate threshold: 60), PROMIS sleep disturbance (version 8b) (T-score range: 28.9–76.5; mild-to-moderate threshold: 60),^3,51^ and the Oswestry Disability Index (ODI).^14^ The PROMIS Depression measures negative mood, views of self, social cognition, and decreased positive affect and engagement. The PROMIS Sleep Disturbance measures perceptions of sleep quality, sleep depth, and any perceived difficulties related to getting and staying asleep. The ODI measures how low back pain affects one's ability to manage in everyday life (version 2.1b) (range: 0–100; a score of 0–20 is considered minimal disability, 21–40 is moderate disability, and 40+ is severe disability).^14^ The number of VR experiences completed during the 56-session therapy (and during the 56-session on-demand period when relevant) was determined once the device was returned to the manufacturing facility.

The Skills-Based VR program is a 56-session self-administered behavioral pain relief skills VR program for cLBP. The program is multimodal and integrates evidence-based skills such as diaphragmatic breathing, biofeedback, cognition and emotion regulation, mindfulness, and pain education into a 56-session therapeutic journey. The program involves daily immersive experiences (2–16 minutes in duration) organized into 8 themes, with content falling into one of the 5 content categories: diaphragmatic breathing, pain education, pain distraction, relaxation/interoception, or mindful escape. The program uses interactive biodata-enabled therapeutics that capture user respiratory rate by an embedded microphone with algorithms synchronizing user data into 3D visual displays and auditory biofeedback.

In adherence with VR-CORE clinical trial guidelines, the Sham was active and composed of nonimmersive, 2D visual content.^26^ Both Skills-Based VR and Sham content were delivered through the Pico G2 4K, and the participants were told the device was delivering VR treatment. Sham offers a form of focused attention on nature scenes and restricted vision on a display that is like a large-screen television. Content included 20 rotating nature videos overlaid with music that were devoid of pain management skills, experiences, or practices. The session duration ranged from 2 to 16 minutes. All packaging and directions were identical across Skills-Based VR and Sham. Participants in both groups were instructed to complete 1 treatment session daily for 56 days.

### 2.3. Statistical analysis

The statistical analysis was divided into 3 sections. The principal analysis focused on the primary endpoint measures of pain intensity and pain interference reduction from baseline to 12 months posttreatment across the Skills-Based VR and Sham groups. Kolmogorov–Smirnov and Shapiro–Wilk tests of sample distribution normality were conducted to determine whether parametric or nonparametric tests were in order. Because 2 endpoint measures were examined, we used a multiple-comparisons corrected *P*-value of 0.025 for each analysis. Sample mean values, SDs, effect sizes, and the *P*-value for the appropriate (parametric or nonparametric) test were reported. The secondary analysis focused on participant-level responder rates. Specifically, a responder was defined as a participant who achieved a clinically meaningful 2-point reduction from baseline to 12 months posttreatment in pain intensity, pain interference, or both. The percentage of responders in each group was examined, as well as the average pain reduction for responders in each group, where the pain reduction for each participant was defined as the average of their pain intensity and pain interference reduction. The tertiary analysis focused on the secondary endpoint measures of PROMIS sleep disturbance, PROMIS depression, and ODI reduction from baseline to 12 months posttreatment across the Skills-Based VR and Sham groups. We determined whether parametric or nonparametric tests were in order; we used the multiple-comparisons corrected *P*-value of 0.017 (0.05/3 = 0.017) and reported sample mean values, SDs, and effect sizes. Mean values, SDs, and effect sizes for pain intensity and pain interference reductions from baseline to 1, 2, 3, 6, and 12 months posttreatment are displayed in Table A-1, http://links.lww.com/PR9/A241 and the associated responder rates are displayed in Table A-2, http://links.lww.com/PR9/A241. Analogous summary statistics for PROMIS sleep disturbance, PROMIS depression, and ODI are displayed in Table A-3, http://links.lww.com/PR9/A241. The data in Tables A-1, A-2, and A-3, http://links.lww.com/PR9/A241, are included for completeness but are not the focus of this report and will not be discussed further.

## 3. Results

As outlined above, the total number of VR experiences completed was low during the on-demand period for the 2 on-demand subgroups (Skills-Based VR: 11.0 ± 14.6; Sham: 10.4 ± 14.5) and did not differ across Skills-Based VR and Sham (*P* = 0.62). Thus, this report combined the 2 Skills-Based VR arms and the 2 Sham arms.

Complete demographics and baseline clinical measures, as well as full results for the primary and secondary endpoints at end-of-treatment, are published and accessible by open source.^31^ Demographics and baseline clinical measures for both groups are also included in Table A-4, http://links.lww.com/PR9/A241. Briefly, the N = 1093 mITT sample was 72% women, 50.8 years average age, with broad racial and ethnic diversity. No significant demographic group differences were observed except the proportion of women (proportion of women: Skills-Based VR group = 0.77; Sham VR = 0.68; *P* = 0.006). Baseline clinical measures suggest a diverse sample with baseline pain intensity, interference, sleep disturbance, and disability in the moderate-to-severe range, and depression as mild. No significant baseline clinical measure differences were observed.

The Skills-Based VR group demonstrated an average pain intensity reduction of 2.0 ± 1.9 points (baseline = 6.6, end-of-treatment = 4.6) and an average pain interference reduction of 2.3 ± 2.0 points (baseline = 6.2; end-of-treatment = 3.9) at end-of-treatment, both significantly larger than for the Sham group.^31^ In addition, 58% of Skills-Based VR participants achieved a 2-point reduction in pain intensity, pain interference, or both. Finally, the Skills-Based VR group demonstrated a significantly larger reduction in the PROMIS Depression, PROMIS Sleep Disturbance, and ODI than the Sham group at end-of-treatment.^31^

Posttreatment survey completion was high with 954 (99%; 1 month post), 929 (97%; 2 months post), 913 (95%; 3 months post), 893 (93%; 6 months post), and 839 (88%; 12 months post) participants who completed the end-of-treatment surveys completing posttreatment surveys. No differences in demographic or baseline clinical variables were observed between Skills-Based VR and Sham VR dropouts or between dropouts and responders within the Skills-Based VR or Sham VR groups.

### 3.1. Group differences: pain intensity and pain interference reduction 12 months posttreatment relative to baseline for skills-based virtual reality vs sham groups

**Figure 1.**
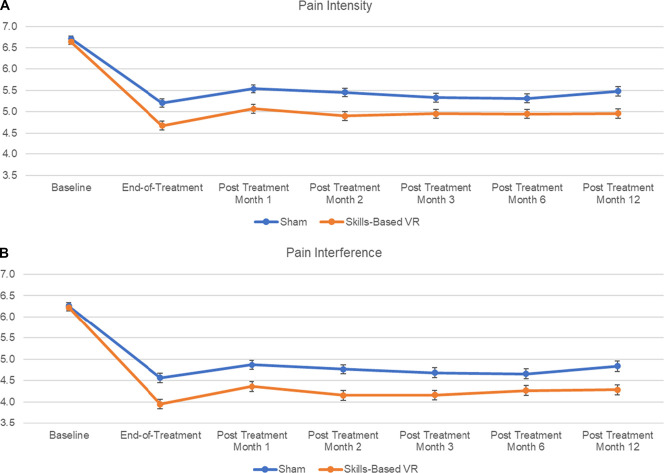
Skills-based VR and Sham VR groups from baseline to 12-months after treatment for (A) BPI pain intensity and (B) BPI pain interference. The x-axis represents time, the y-axis represents pain rating on a 0 to 10 scale. Standard error bars included. BPI, Brief Pain Inventory; VR, virtual reality.

Table 1 displays the pain intensity and pain interference reduction results at 12 months posttreatment relative to baseline, and for illustrative purposes, Figure 1 displays the group average results over time for BPI pain intensity and pain interference from baseline to 1, 2, 3, 6, and 12 months posttreatment. Sample distribution test results converged in suggesting that the pain intensity and pain interference reduction sample distributions for Skills-Based VR and Sham violated normality (all *P* < 0.005). Thus, we applied Mann–Whitney *U* tests and found that the pain intensity and pain interference reduction distributions differed significantly across Skills-Based VR and Sham groups (both *P* < 0.001). (Independent-group *t*-tests were also conducted and the results converged with those from the Mann–Whitney U test in showing that the pain intensity [*P* = 0.0001] and pain interference [*P* < 0.001] reductions were larger for the Skills-Based VR group than the Sham group.) At 12 months posttreatment, BPI pain intensity reductions were larger for the Skills-Based VR group (1.7 ± 2.1) than for the Sham group (1.2 ± 2.0), and BPI pain interference reductions were larger for the Skills-Based VR group (1.9 ± 2.3) than for the Sham group (1.3 ± 2.1). The Skills-Based VR group pain intensity and pain interference reductions did not reach the clinically meaningful 2-point threshold but approached clinical meaningfulness.^27^

**Table 1:** Mean (and SD) Brief Pain Inventory pain intensity and interference, Patient-Reported Outcomes Measurement Information System sleep disturbance, Patient-Reported Outcomes Measurement Information System depression, and the Oswestry Disability Index reductions for skills-based virtual reality and sham at 12 months posttreatment relative to baseline.

	BPI pain intensity	BPI pain interference	PROMIS sleep disturbance	PROMIS depression	Oswestry Disability Index
Mean (SD) point reduction from baseline to 12 months posttreatment					
Skills-based VR	1.7 (2.1)	1.9 (2.3)	5.2 (8.1)	1.7 (9.3)	10.0 (15.5)
Sham	1.2 (2.0)	1.3 (2.1)	3.3 (7.2)	0.0 (9.0)	6.1 (15.5)
Mann–Whitney U, *P*-value: Skills-based VR vs sham	<0.001	<0.001	0.002	0.02	0.001
Effect size: skills-based VR vs sham	0.23	0.22	0.23	0.09	0.14

BPI, Brief Pain Inventory; PROMIS, Patient-Reported Outcomes Measurement Information System; VR, virtual reality.

### 3.2. Responder analysis: clinically meaningful pain reductions at 12 months posttreatment relative to baseline for skills-based virtual reality vs sham participants

Table 2 displays the results from the responder analysis that compared the percentage of participants who achieve a clinically meaningful 2-point reduction from baseline to 12 months posttreatment in pain intensity, pain interference or both for Skills-Based VR and Sham. The average pain reduction (based on the average pain intensity and pain interference reduction for each participant) for responders by study group was also included.^15,27^ Significantly more Skills-Based VR participants (52%) were classified as responders than Sham participants (42%) (*P* = 0.002), and the average pain reduction for the Skills-Based VR group (3.7 ± 1.4) was larger than for the Sham group (3.5 ± 1.2).

**Table 2:** Percentage of skills-based virtual reality and Sham participant responders (ie, participants who achieved a 2-point reduction from baseline to 12 months posttreatment in pain intensity, pain interference, or both) and the average pain reduction (defined as the average pain intensity and pain interference reduction) for responders in each group.

	Percentage of responders	Mean (and SD) pain reduction for responders
Percentage of participants with 2+ point reduction		
Skills-based VR	52	3.7 (1.4)
Sham	42	3.5 (1.2)

VR, virtual reality.

### 3.3. Group differences: Patient-Reported Outcomes Measurement Information System sleep disturbance, Patient-Reported Outcomes Measurement Information System depression, and Oswestry Disability Index reduction 12-months posttreatment relative to baseline for skills-based VR vs sham groups

Table 1 displays the PROMIS sleep disturbance, PROMIS depression, and ODI reduction results at 12 months posttreatment relative to baseline. Sample distribution test results converged in suggesting that the PROMIS sleep disturbance, PROMIS depression, and ODI reduction sample distributions for Skills-Based VR and Sham violated normality (all *P* < 0.005). Thus, we applied Mann–Whitney *U* tests and found that the PROMIS sleep disturbance (*P* = 0.002) and ODI distributions (*P* = 0.001) differed significantly across Skills-Based VR and Sham groups, whereas the PROMIS depression distribution test (*P* = 0.02) did not reach the *P* = 0.017 multiple-comparisons threshold. At 12 months posttreatment, PROMIS sleep disturbance reductions were larger for the Skills-Based VR group (5.2 ± 8.1) than for the Sham group (3.3 ± 7.2) (*P* = 0.002), ODI reductions were larger for the Skills-Based VR group (10.0 ± 15.5) than for the Sham group (6.1 ± 15.5) (*P* = 0.001), whereas PROMIS depression reductions were larger for the Skills-Based VR group (1.7 ± 9.3) than for the Sham group (0 ± 9.0), although this difference did not reach statistical significance.

## 4. Discussion

The purpose of the present report was to examine the long-term effectiveness at 12 months posttreatment relative to baseline for a double-blinded, placebo-controlled, randomized controlled trial comparing a 56-session self-administered, in-home Skills-Based VR for Chronic Pain program with Sham VR in a cLBP sample that was large, demographically diverse, and clinically severe. The Skills-Based VR group was superior to the Sham group in reducing pain intensity and pain interference at 12 months after treatment with average pain reductions of 1.7 points (intensity) and 1.9 points (interference). Although these average reductions are clinically meaningful based on a 1.5-point MCID,^6,8,27^ we applied the more stringent 2-point MCID. More than half of the Skills-Based VR participants (52%) maintained at least a 2-point reduction in pain intensity, pain interference, or both at 12 months posttreatment. These results are comparable with those from a previous study^19,21,30^ that included a relatively homogeneous sample of moderate clinical severity. In the present study, the 12-month average pain intensity and interference reductions of 1.7 and 1.9, respectively, are comparable and even slightly greater than the pain reductions observed in the study by Garcia et al. at 6 months (intensity = 1.5, interference = 1.9).^19^

Long-term effectiveness at 12 months posttreatment approached clinical meaningfulness for Skills-Based VR, yet the strong Sham outcomes resulted in small between-group effect sizes (pain intensity = 0.23, pain interference = 0.22). Strong placebo effects for active controls are well established in the literature,^2,18^ and the Sham in this study adhered to elements recommended by expert consensus for rigor in clinical research. In particular, the active VR Sham involved a novel device with strong motivational pull. Although not designed to be therapeutic, the active Sham used content in the form of pain distraction and relaxation that has therapeutic value.^23^ Despite this strong active control, the Skills-Based VR program evidenced statistically larger reductions in pain intensity and pain interference that approached clinical meaningfulness.

Questions remain about the mechanisms of action for a 56-session VR therapy program and how long-term pain relief is achieved 12 months after treatment was stopped. We offer a few hypotheses on treatment mechanisms. First, the Skills-Based VR program helps patients develop self-regulatory skills that they can use in their daily lives, outside the VR headset, and ongoingly once the 56-session program is complete. In other words, the device is a learning tool designed to equip individuals with lasting pain relief skills. Second, immersive content delivered in VR broadly engages multiple centers in the brain in synchrony,^29,32^ and this may be explanatory for why we see suggestion of stronger learning effects for Skills-Based VR for chronic pain than other modalities that are not 3D immersive.^7^ Third, the content of the program is consistent, quality controlled, and includes deliberate elements of content repetition to reinforce clinical messages, user responses, and skills entrainment. By contrast, other VR pain solutions applied in different contexts, such as for acute pain management, may use distraction for momentary analgesia without attention to self-regulatory skills building that is important for long-term relief in the chronic pain context. Direct comparison of the Skills-Based VR program with other VR therapies (reviewed elsewhere^36,38^) may offer a fruitful future direction. Even so, a direct comparison of Skills-Based VR with an audio-only version of CBT has been conducted and Skills-Based VR was found to be superior.^7^

### 4.1. Limitations

The following limitations bear consideration when evaluating the study results: (1) self-reported cLBP was not confirmed by healthcare professionals, (2) the treatment was adjunctive so additional treatments during VR therapy and during the 12-month posttreatment phase could affect the results (although group randomization should minimize or eliminate between-group disparity in receipt of additional treatments), (3) a focus on cLBP and did not examine other chronic pain conditions, and (4) lack of other arms (eg, a wait-list control).^36,38^

## 5. Conclusions

Community-based clinicians continue to struggle in their quest for nonpharmacologic options to treat cLBP. Nonpharmacologic behavioral interventions exist, but barriers to access are prevalent and long-term treatment effectiveness is variable. Results of this study suggest that an in-home VR-pain relief skills-based therapy can provide consistent, quality controlled, easily accessible cLBP treatment that maintains clinical effectiveness 12 months posttreatment, and approached clinical meaningfulness.

## Disclosures

Dr. Maddox, Dr. Oldstone, Roselani Maddox, Kelsey Ffrench, and Takisha Adair are employees of AppliedVR, Inc. Joshua Sackman is president of AppliedVR, Inc. Dr. Sparks is a former employee of AppliedVR, Inc who was employed during execution of the study. Dr. Darnall is chief science advisor for AppliedVR, Inc. Dr. Darnall has authored or coauthored 5 pain treatment books for patients and clinicians and receives royalties for 4. Dr. Darnall is the principal investigator for pain research grants and awards from the National Institutes of Health (NIH) and the Patient-Centered Research Outcomes Research Institute (none specific to the current work). Dr. Darnall is a co-investigator on 2 NIH research grants investigating virtual reality analgesia; neither of these grants is specific to the current work. Dr. Darnall serves on the Board of Directors for the American Academy of Pain Medicine and is on the Board of Directors for the Institute for Brain Potential. Dr. Darnall is a scientific member of the NIH Interagency Pain Research Coordinating Committee, the Centers for Disease Control and Prevention (CDC) Opioid Workgroup (2020–2021), and the Pain Advisory Group of the American Psychological Association.

## Supplementary Material

SUPPLEMENTARY MATERIAL

## Supplemental digital content

Supplemental digital content associated with this article can be found online at http://links.lww.com/PR9/A241.
