# Supplementary material for: Twelve-month results for a randomized sham-controlled effectiveness trial of an in-home skills-based virtual reality program for chronic low back pain
Source: Pain Rep. 2024 Sep 4;9(5):e1182. doi: 10.1097/PR9.0000000000001182 (PMC11377093; doi:10.1097/PR9.0000000000001182)
Supplement: SUPPLEMENTARY MATERIAL [file painreports-9-e1182-s001.pdf]

Appendix

Table A-1. Mean (and standard deviation) BPI Pain intensity and interference reductions for Skills-Based VR and Sham at 1, 2, 3, 6, and 12-months post-treatment relative to baseline.

		Months Post-Treatment				
		1	2	3	6	12
BPI Pain Intensity						
Average Point Reduction from Baseline (standard deviation)	Skills-Based VR	1.6 (1.9)	1.7 (2.0)	1.7 (2.1)	1.7 (2.2)	1.7 (2.1)
	Sham	1.2 (1.8)	1.2 (1.8)	1.4 (1.8)	1.4 (1.8)	1.2 (2.0)
Effect Size: Skills-Based VR vs Sham		0.22	0.25	0.17	0.17	0.23
BPI Pain Interference						
Average Point Reduction from Baseline (standard deviation)	Skills-Based VR	1.9 (2.0)	2.0 (2.0)	2.0 (2.1)	1.9 (2.3)	1.9 (2.3)
	Sham	1.3 (1.9)	1.4 (1.8)	1.5 (1.9)	1.5 (1.9)	1.3 (2.1)
Effect Size: Skills-Based VR vs Sham		0.21	0.25	0.26	0.16	0.22

Table A-2. Percentage of Skills-Based VR and Sham participant responders (i.e., participants who achieved a 2-point reduction from baseline to 1, 2, 3, 6, or 12-months post-treatment in pain intensity, pain interference or both) and the average pain reduction (defined as the average pain intensity and pain interference reduction) for responders in each group.

		Months Post-Treatment				
		1	2	3	6	12
Percentage of Participants with 2+ Point Reduction	Skills-Based VR	50	55	52	52	52
	Sham	41	42	43	45	42
Average Pain Reduction from Baseline (standard deviation)	Skills-Based VR	3.4 (1.1)	3.5 (1.3)	3.6 (1.4)	3.6 (1.4)	3.7 (1.4)
	Sham	3.2 (1.0)	3.2 (1.1)	3.3 (1.1)	3.3 (1.2)	3.5 (1.2)

Table A-3. Mean (and standard deviation) PROMIS sleep disturbance, PROMIS depression, and the Oswestry Disability Index reductions for Skills-Based VR and Sham at 1, 2, 3, 6, and 12-months post-treatment relative to baseline

		Months Post-Treatment				
		1	2	3	6	12
PROMIS Sleep Disturbance						
Average Point Reduction from Baseline (standard deviation)	Skills-Based VR	4.3 (7.0)	4.6 (7.5)	4.6 (8.1)	4.6 (7.9)	5.2 (8.1)
	Sham	2.5 (6.5)	3.0 (6.8)	3.2 (7.1)	3.0 (7.0)	3.3 (7.2)
Effect Size: Skills-Based VR vs Sham		0.24	0.21	0.16	0.20	0.23
PROMIS Depression						
Average Point Reduction from Baseline (standard deviation)	Skills-Based VR	1.6 (8.0)	1.7 (8.4)	1.6 (8.8)	1.5 (8.8)	1.7 (9.3)
	Sham	-.005 (7.7)	0.6 (8.0)	0.7 (8.0)	0.5 (8.0)	0.0 (9.0)
Effect Size: Skills-Based VR vs Sham		0.12	0.06	0.03	0.01	0.09
Oswestry Disability Index						
Average Point Reduction from Baseline	Skills-Based VR	8.3 (12.6)	9.4 (13.5)	9.1 (14.0)	9.7 (14.7)	10.0 (15.5)
	Sham	4.9 (13.3)	6.9 (13.4)	6.8 (13.3)	6.3 (12.9)	6.1 (15.5)
Effect Size: Skills-Based VR vs Sham		0.14	0.10	0.09	0.13	0.14

Table A-4. Participant demographics and baseline clinical measures from Maddox et al (2023)

	Skills-Based VR (n=536)	Sham (n=531)	*P*
**Gender**			
Female	411 (76.7)	361 (68)	0.006
Male	124 (23.1)	169 (31.8)	
Non-binary	1(0.2)	1 (0.2)	
**Race and Ethnicity**			0.46
American Indian/Alaska Native	4 (0.7)	5 (0.9)	
Asian/Pacific Islander	12 (2.2)	16 (3.0)	
Black/African American	81 (15.1)	95 (17.9)	
Caucasian	370 (69.0)	351 (66.1)	
Hispanic/Latin	18 (3.4)	10 (1.9)	
Multiracial	51 (9.5)	54 (10.2)	
**Age (y)**	50.4 (13.5)	51.1 (128)	0.35
**Household Annual Income**			0.26
<$10,000	40 (7.5)	44 (8.3)	
$10,000 - $19,999	75 (14)	57 (10.7)	
$20,000 - $29,999	52 (9.7)	60 (11.3)	

$30,000 - $39,999	56 (10.4)	58 (10.9)	
$40,000 - $49,999	51 (9.5)	46 (8.7)	
$50,000 - $59,999	40 (7.5)	44 (8.3)	
$60,000 - $69,999	31 (5.8)	40 (7.5)	
$70,000 - $79,999	46 (8.6)	63 (11.9)	
≥ $80,000	145 (27.1)	119 (22.4)	
Body mass index	31.3 (7.9)	31.7 (8.4)	0.34
BPI Pain Intensity	6.6 (1.5)	6.7 (1.5)	0.35
BPI Pain Interference	6.2 (1.8)	6.2 (1.8)	0.87
PROMIS Anxiety	56.1 (9.1)	55.6 (9.5)	0.39
PROMIS Sleep Disturbance	60.7 (7.1)	60.8 (7.3)	0.78
PROMIS Depression	54.9 (9.3)	54.6 (8.9)	0.50
Oswestry Disability Index (ODI)	41.5 (15.6)	41.0 (16.9)	0.61

Note: Data are presented as n (%) or mean (SD). P-value from Chi-squared (or Fisher's exact) test comparing Skills-Based VR to Sham
